# Transcriptome Analysis of Acetyl-Homoserine Lactone-Based Quorum Sensing Regulation in *Yersinia pestis*

**DOI:** 10.1371/journal.pone.0062337

**Published:** 2013-04-19

**Authors:** Christopher N. LaRock, Jing Yu, Alexander R. Horswill, Matthew R. Parsek, F. Chris Minion

**Affiliations:** 1 Department of Veterinary Microbiology and Preventive Medicine, Iowa State University, Ames, Iowa, United States of America; 2 Department of Microbiology, University of Washington, Seattle, Washington, United States of America; 3 Department of Microbiology, Roy J. and Lucille A. Carver College of Medicine, University of Iowa, Iowa City, Iowa, United States of America; University Medical Center Utrecht, Netherlands

## Abstract

The etiologic agent of bubonic plague, *Yersinia pestis,* senses self-produced, secreted chemical signals in a process named quorum sensing. Though the closely related enteric pathogen *Y. pseudotuberculosis* uses quorum sensing system to regulate motility, the role of quorum sensing in *Y. pestis* has been unclear. In this study we performed transcriptional profiling experiments to identify *Y. pestis* quorum sensing regulated functions. Our analysis revealed that acyl-homoserine lactone-based quorum sensing controls the expression of several metabolic functions. Maltose fermentation and the glyoxylate bypass are induced by acyl-homoserine lactone signaling. This effect was observed at 30°C, indicating a potential role for quorum sensing regulation of metabolism at temperatures below the normal mammalian temperature. It is proposed that utilization of alternative carbon sources may enhance growth and/or survival during prolonged periods in natural habitats with limited nutrient sources, contributing to maintenance of plague in nature.

## Introduction

Plague, or the Black Death, has killed over two hundred million people and remains the most devastating epidemic bacterial disease. Plague, caused by *Yersinia pestis*, is a zoonotic disease primarily spread between mammals by fleas. When a flea bites an infected rodent, *Y. pestis* is ingested with the blood meal. After a period of growth in the flea gut, transmission from the flea results from colonization of the proventricular valve, impairing valve function and allowing backflow of infectious blood into subsequently bit mammals. *Yersinia pestis* differentiated 2,600 to 28,000 years from *Y. pseudotuberculosis* [1], which causes mild enteritis in humans. With astonishingly few genetic changes, *Y. pestis* gained a dramatically enhanced ability to induce mortality in mammalian hosts, and it acquired the ability to colonize an additional host, the flea. Thus, as a parasite of both mammals and fleas, it has adapted to grow in two very different environments.

Pathogens exploit the host as a growth medium, and nutrient availability can affect tissue tropism, the duration of infections, and the expression of virulence factors [2]. During the evolution of *Y. pestis*, there probably has been a strong selective pressure to efficiently utilize the carbon and energy sources present in blood meals, since the flea is a closed environment that could rapidly become nutritionally limiting. Fleas are a long-term reservoir for *Y. pestis* and can remain infected for periods longer than a year [3]. The feeding frequency and the nutritional quality of each blood meal can both influence growth, and ultimately, the long-term viability of *Y. pestis* [4]. In the model of early phase transmission, *Y. pestis* may be fittest when it grows quickly on readily metabolized substrates [5], though complete catabolization of the blood meal leading to flea starvation could prevent transmission because of the high fitness cost to the flea [6]. Additionally, *Y. pestis* can survive in soil for at least 24 days and possibly other natural environments as well [7]. Nutrient availability can vary significantly in these environments.

Quorum sensing (QS) is a process by which bacteria can coordinately regulate gene expression in response to the sensing of diffusible chemical signals. There are two different types of signaling systems, autoinducer-2 (AI-2), which is widely conserved among a diverse phylogenetic range of bacterial species and involves the sensing of furanosyl ester signals, and acyl-homoserine lactone (AHL) QS. In AHL QS, which is primarily found in the Gram negative Proteobaceteria, the AHL acyl chain can vary in length and degree of substitution depending upon the species.

Quorum sensing regulates flagellar motility and other functions essential to enteric infection in *Y. pseudotuberculosis,* a related species [8]–[10]. *Yersinia pestis* has three conserved, intact QS systems, a highly conserved AI-2/LuxS system and two LuxI-LuxR AHL systems. Yet *Y. pestis* is amotile [11], suggesting that QS regulates distinct functions in this species. The two AHL systems in *Y. pestis* are designated as YpeIR and YspIR (YtbIR and YpsIR in the strain KIM nomenclature) and are unlinked in the chromosome. Previous work suggests that QS is not involved in mouse virulence or the ability to colonize and block fleas for short durations [12], so the function, if any, QS serves for *Y. pestis* remains a mystery.

This study utilized a transcriptional profiling approach to identify QS-regulated functions. QS was found to control expression of genes involved in the catabolism of alternative carbon sources. Regulation of these functions occurred in a temperature dependent manner, suggesting a role for QS in carbon utilization during prolonged periods of nutrient limitation in a manner that may maximize bacterial growth and long-term survival leading to maintenance in the wild.

## Materials and Methods

### Bacterial Strains, Plasmids, and Media

All bacterial strains used in this study are listed in Table 1. The *Y. pestis* strains R88, R114 and R115 were constructed by Robert Perry (University of Kentucky) for our studies using recombineering plasmids previously used to construct the same mutations in strain KIM [13]. Cultures of *Yersinia* were routinely grown in Heart Infusion Broth (HIB) at 30°C with aeration or on plates containing 1% HIB, 2% Agar, 0.01% Congo Red, and 0.2% galactose [14].

**Table 1:** Strains and plasmids.

Name	Description	Source
R88	*Y. pestis* CO92 Δ*pgm* pCD^+^ pMT1^+^ pPCP+	R. Perry (Univ. Kentucky)
R114	*Y. pestis* CO92 Δ*pgm* pCD^+^ pMT1^+^ pPCP^+^ Δ*ypeIR* Δ*yspIR*	R. Perry
R115	*Y. pestis* CO92 Δ*pgm* pCD^+^ pMT1^+^ pPCP^+^ Δ*ypeIR* Δ*yspIR*, *luxS*::Kan	R. Perry
ISM1980	*Y. pestis* CO92 Δ*pgm* pCD^+^ pMT1^+^ pPCP^+^ *luxS*::Kan	G. Phillips (Iowa State University)
MM32	*Vibrio harveyi* Δ*luxN* Δ*luxS*	M. Parsek
pYspIR	*Y. pestis* CO92 *yspIR* operon cloned into pTrc99a MCS	This study
pYpeIR	*Y. pestis* CO92 *ypeIR* operon into pTrc99a MCS	This study

### Plasmid Constructions

The genes *yspIR* and *ypeIR* were directionally cloned into pTrcHis by amplifying them by PCR, digesting the product with *Kpn*I and *Xba*I, and ligating into digested, dephosphorylated pTrcHis. Primers used for amplification are described in Table S1. The plasmids were transformed into the appropriate *Y. pestis* strain by electroporation.

### Bioassay for AI-2 Production

Production of AI-2 by *Yersinia* sp. was assayed with a *Vibrio harveyi* bioreporter that is luminescent proportional to exogenous AI-2 levels. The procedure used was similar to previous assays [15].

### AHL Detection

The base medium for AHL assays was Thoroughly Modified Higachi’s (TMH) media [16]. The methionine concentration was lowered to 100 µM, making methionine limiting late during growth (data not shown), and allowing rapid incorporation of the radiolabel. Cultures were grown in 5 ml of methionine-modified TMH at 30°C with shaking to an OD_600_ of 1.6. Labeling and identification of AHLs was essentially as previously [17]. Labeled AHLs were produced during an 1.5 h incubation after the addition of 5 µCi of [*carboxy* ^−14^C]-methionine (American Radiochemical). Supernatants were removed from cell pellets, organics were extracted from the supernatants with acidified ethyl acetate, and samples were dried under N_2_ gas. The extracts were finally eluted in 200 µl of 50% methanol and separated on a C_18_ reversed-phase column over a 10%–100% methanol gradient by high-performance liquid chromatography (HPLC). Seventy 1-ml fractions were collected, to which 4 ml of scintillation cocktail 3a70B (Research Products) was added, and radioactivity was determined by scintillation counting. AHL peaks were identified by comparison to internal controls.

### Microarray Studies

**Figure 1:**
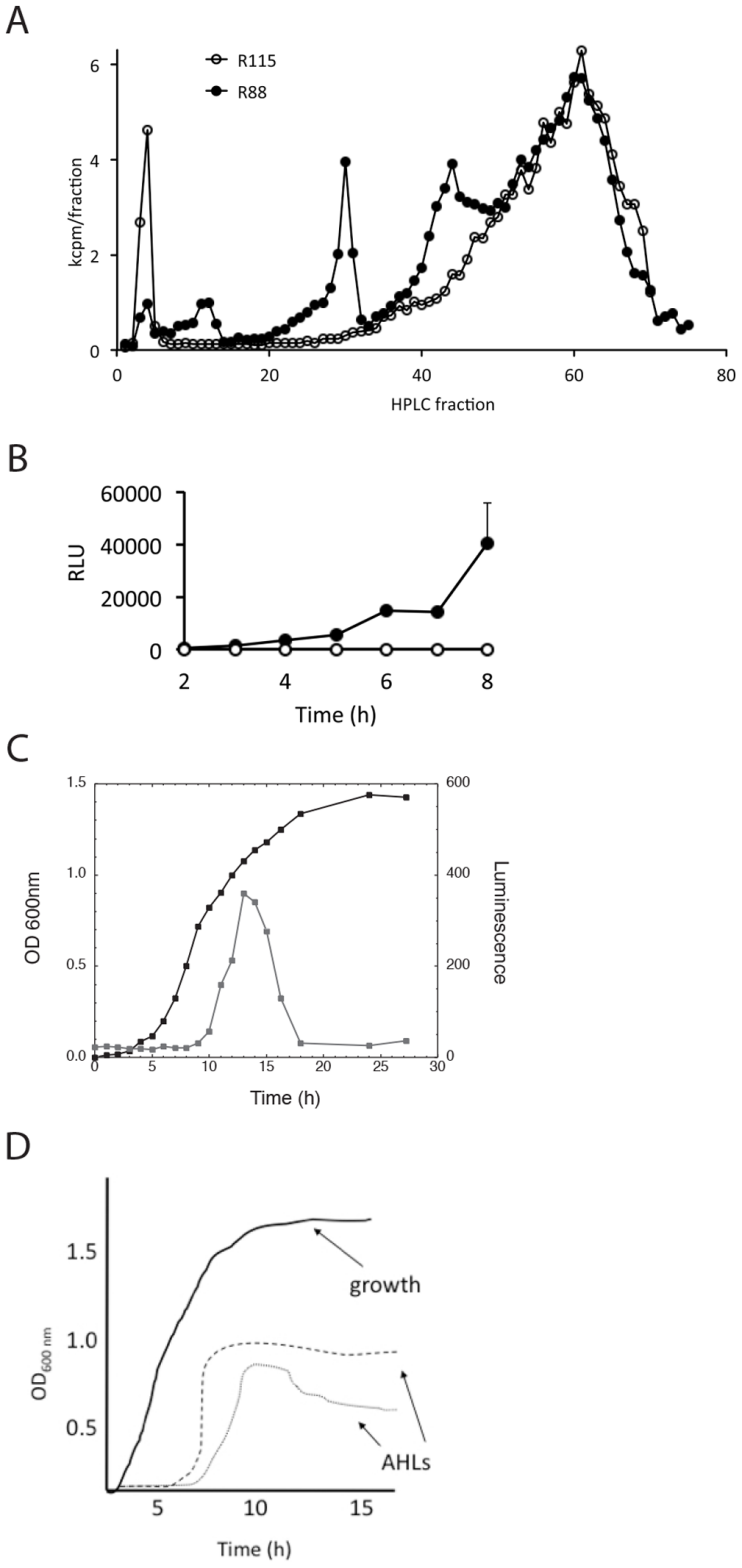
Identification of the *Y. pestis* quorum-sensing molecules. (A) HPLC fractionation profiles of ^14^C-labeled AHL produced by R88 *Y. pestis* (solid circles) compared to R115 QS^-^ *Y. pestis* (empty circles). The peaks absent from organic extracts of R115 *Y. pestis* supernatants correspond to C8-, C6-, and oxo-C6-AHL. (B) AI-2 production during the growth of R88 *Y. pestis* (solid circles) and R115 (empty circles) was monitored by adding the cell-free supernatants at the indicated time points to a *V. harveyii* reporter strain that is bioluminescent (RLU) in response to AI-2. Data are representative of at least three independent studies. (C and D) The production of AI-2 (C) and AHL (D) signals as a function of growth.

Microarray studies were performed as described previously [18] by comparing strains R88 and R114 grown at a mid logarithmic phase of growth. Briefly, an overnight culture was diluted 1∶100 and grown for 9 h at 30°C in BHI broth at which time an optical density 600 nm of 1.0 is reached. At this optical density, the cells are in full quorum sensing mode (Fig. 1). RNAprotect Bacterial Reagent (Qiagen) was added to cell suspensions at a 2∶1 volume ratio of reagent to culture. After 2 min, the cells were pelleted and the pellets were snap frozen and stored at −70°C until the RNA was isolated. RNA was extracted from frozen cell pellets using the Trizol (Ambion) reagent protocol. The RNA was treated with DNase I (Ambion) at 37°C for 30 min to remove contaminating genomic DNA. The RNA was purified and concentrated by Microcon Ultracel/YM 30. Target generation was performed as described [19].

Corresponding equal amounts (1.5 µg) of Alexa 555- or Alexa 647-labelled cDNA targets were mixed and dried using a Thermo Scientific Savant DNA SpeedVac Concentrator. Targets were suspended in 60 µl hybridization buffer (40% formamide, 5x SSC, 0.1% SDS, 0.6 µg/µl Salmon Sperm DNA), incubated at 95°C for 5 min, centrifuged (10,000×*g*, 4 min), and kept at room temperature until placed in a hybridization chamber. Hybridization lasted for 16 h at 42°C and washed in a series of wash buffers (2x saline-sodium citrate (SSC) −0.1% SDS; 0.1x SSC –0.1% SDS, and 0.1x SSC) and dried by centrifugation at 1500×*g* for 30 s.

Six RNA samples from independent (biological replicates) *Y. pestis* CO92 R114 AHL deficient cultures were paired with six RNA samples from independent control *Y. pestis* CO92 R88 cultures for hybridization to six two-color microarrays. The microarrays consisted of the same oligonucleotide set previously described [20] printed to Corning epoxide slides. For three arrays, the control RNA sample was labeled with Alexa 555 dye and the experimental RNA sample was labeled with Alexa 647 dye; the dyes were reversed for the other three arrays to account for any dye bias.

Data acquisition, normalization and data analysis have been described [21]. Briefly, following scanning of each dye channel three times under varying laser power and PMT gain settings with a ScanArray Express laser scanner (Applied BioSystems, Inc., Foster City, Calif.) [22], the images were quantified using the softWorRx Tracker analysis software package (Applied Precision, Inc., Issaquah, Wash.). Following correction for local background, an adjustment was made to the natural logarithm of the background-corrected signals, and the median across the multiple scans was computed for each spot to obtain one value for each combination of spot and dye (Alexa 555 versus 647) channel. These data were then subjected to LOWESS normalization to adjust for intensity-dependent dye bias [23], [24], and the normalized values for duplicate spots were averaged within each array to produce one normalized measure of expression for each of the probe and RNA samples.

A separate mixed linear model analysis was conducted for each probe sequence using the normalized data [25]. Each mixed model included fixed effects for treatment and dye (Alexa 555 versus 647) as well as random effects for slide and slide-by-region interaction. A *t*-test for differential expression across treatments was conducted for each probe, and the *p*-values from these *t*-tests were converted to *q*-values using the method of Storey and Tibshirani [26]. These *q*-values were used to obtain approximate control of the False Discovery Rate (FDR) at a specified value (a *q* value of 0.05 would have a corresponding FDR value of 5%). Along with *q*-values, estimates of fold-change were computed for each probe by taking the inverse natural log of the mean treatment difference estimated as part of our mixed linear model analyses.

The microarray data can be accessed from the Gene Expression Omnibus (GEO) using series accession number GSE29240 (http://www.ncbi.nlm.nih.gov/geo/).

### qRT-PCR

Significant transcriptional differences demonstrated by the microarrays between the treatment and control cultures were verified by qRT-PCR. Eight up-regulated genes in R88 (*aceA, galT, mglABC*, *malE, lamB, katY*) were chosen for confirmation based on the magnitude of transcriptional change. The gene *leuB* was used as a control for the RT reactions because it did not show significant changes in this study. The qRT-PCR was performed by using the SensiMix™ SYBR No-Rox One-Step kits (Bioline) following the manufacturer's protocol. Cycling was performed on the Mx3005P (Stratagene). Data were analyzed according to Gallup & Ackermann [27]. The primers used are described in Table S1.

For analysis of effects of QS mutations on key metabolic genes, qRT-PCR was performed as follows. cDNA was amplified from purified RNA using random primers, as per the manufacturer’s protocol (Im-Prom II reverse transcription system, Promega). Specific transcripts were verified with PCR primers designed using Primer3 software and ordered from Operon (Table S1). Data is expressed as the Log_2_ of transcript differences between each mutant and that of WT, normalized to the 16 S rRNA of each sample.

### Determination of Growth Rates

Growth rates for the utilization of a substrate as a carbon and energy source was determined in modified TMH, with gluconate removed as a carbon and energy source [28]. To require greater stringency on single carbon source utilization, the media was additionally modified to remove non-essential amino acids. Isoleucine, valine, phenylalanine, methionine, and glycine were added to concentrations used previously [29]. The HEPES concentration was increased to 50 mM, and media buffering capacity was increased by the addition of 100 mM PIPES. To this base media, maltose, acetate, galactose, glucose, sucrose, gluconate, succinate or citrate were added to 0.2% and the pH adjusted to 7.4. Growth was measured using a Bioscreen C microplate reader (MTX LabSystems, Inc).

### Differentiation between Oxidative and Fermentative Metabolism

Assays were carried out similarly to standard methods [30]. The medium contained 0.2% peptone, 0.1% yeast extract, 0.5% sodium chloride, 0.03% dibasic potassium phosphate, 0.3% agar, and 0.001% Congo red. After autoclaving, filter sterilized carbohydrates were added to 1% from stock solutions. Sterile borosilicate culture tubes were filled with 5 ml of media and subsequently allowed to solidify. Cultures were inoculated by stabbing fermentative tubes, which were then covered with 1 ml of sterile, melted petroleum jelly.

## Results

### *Y. pestis* CO92 Produces Both AHL and AI-2 QS Signals

Bioreporter and thin-layer chromatography experiments have previously identified AI-2 signaling and 3-oxo-C6-, C6-, and C8- AHL signaling molecules for a *Medievalias* biovar of *Y. pestis* [13]. To determine if CO92, an isolate of the North American-endemic biovar *Orientalis*, produces similar signals, a radiolabeling assay was employed using a Δ*pgm* mutant of CO92 (Fig. 1A) [17]. The three major peaks eluted in fractions 11–12, 30 and 45, correspond to 3-oxo C6, C6 and C8 AHLs, respectively [17]. This supports previously published work that demonstrated these three compounds as primary AHL signals in *Y. pestis*. As expected, these three peaks were absent in R115, the QS null strain. To demonstrate that AI-2 signaling is also intact in the R88 background, culture supernatants were added to a *Vibrio harveyi* bioreporter assay responsive to exogenous AI-2 [15]. AI-2 dependent bioluminescence was observed in strain R88, but not in ISM1980, the isogenic *luxS* mutant strain. AI-2 production was detected in cultures grown at both 37 and 30°C (Fig. 1B).

### *Y. pestis* Uses AHL-based Quorum Sensing to Regulate Genes Involved in Catabolism of Suboptimal Carbon Sources

The presence of an intact AHL QS system suggested *Y. pestis* has AHL regulated functions. To gain insight into the role of AHL QS in *Y. pestis*, we performed transcriptional profiling experiments to identify quorum sensing regulated genes. Transcript levels were compared in the Δ*pgm* strain R88 and R114, an AHL-null strain, during the logarithmic phase of growth at 30°C in HIB. A total of 335 genes were found to be differentially expressed at least 1.5-fold in *Y. pestis* using cutoff values *p*<0.01 and *q* <0.03. Positively-regulated genes controlled by AHL signaling included those involved in the galactose and maltose metabolic pathways, the *katY*-encoded catalase, and the glyoxylate bypass pathway (Table 2). The remaining significant up- or down-regulated genes (*p*<0.05, *q* <0.08) are listed in Table S2.

**Table 2:** Selected genes significantly up-regulated by QS.

Gene symbol	Gene function		*p* value	*q* value	FC*	qRT-PCR
**Glyoxylate bypass**						
*aceA*		Isocitrate Lyase	0.0000	0.0041	3.95	10.12
*aceB*		Malate Synthase	0.0001	0.0062	3.57	
*aceK*		Bifunctional Isocitrate Dehydrogenase Kinase/Phosphatase Protein	0.0017	0.0128	2.31	
**Galactose**						
*galE*		UDP-Galactose-4-Epimerase	0.0005	0.0078	10.96	
*galK*		Galactokinase	0.0013	0.0119	3.30	
*galT*		Galactose-1-Phosphate Uridylyltransferase	0.0006	0.0091	3.71	7.26
*mglA*		Galactose/Methyl Galaxtoside Transporter ATP-Binding Protein	0.0004	0.0073	4.40	4.57
*mglB*		Galactose-Binding Protein	0.0000	0.0041	8.35	4.27
*mglC*		Beta-Methylgalactoside Transporter Inner Membrane Component	0.0013	0.0119	6.96	3.67
**Maltose operon**						
*lamB*		Maltoporin	0.0010	0.0109	2.26	5.34
*malE*		Maltose ABC Transporter Periplasmic Protein	0.0001	0.0062	6.23	7.64
*malF*		Maltose Transporter Membrane Protein	0.0096	0.0270	4.53	
*malG*		Maltose Transporter Permease	0.0087	0.0255	4.60	
*malK*		Maltose/Maltodextrin Transporter ATP-Binding Protein	0.0002	0.0062	7.60	
*malM*		Maltose Regulon Periplasmic Protein	0.0002	0.0062	5.33	
*malQ*		4-Alpha-Glucanotransferase	0.0002	0.0062	3.58	
*malS*		Periplasmic Alpha-Amylase Precursor	0.0062	0.0216	3.88	
**Detoxification**						
*ahpC*		Putative Alkyl Hydroperoxide Reductase Subunit C	0.0016	0.0125	1.66	
*katY*		Catalase-Peroxidase	0.0001	0.0060	2.96	3.29
*maeB*		Malic Enzyme	0.0023	0.0144	1.90	
*sodB*		Superoxide Dismutase	0.0012	0.0114	1.63	
*sodC*		Superoxide Dismutase [Cu-Zn] Precursor	0.0097	0.0273	1.65	
*tpx*		Thiol Peroxidase	0.0016	0.0125	1.61	

* FC = fold change.

### QS Mutant Strain R114 was Deficient for Growth on Selected Sugars

**Figure 2:**
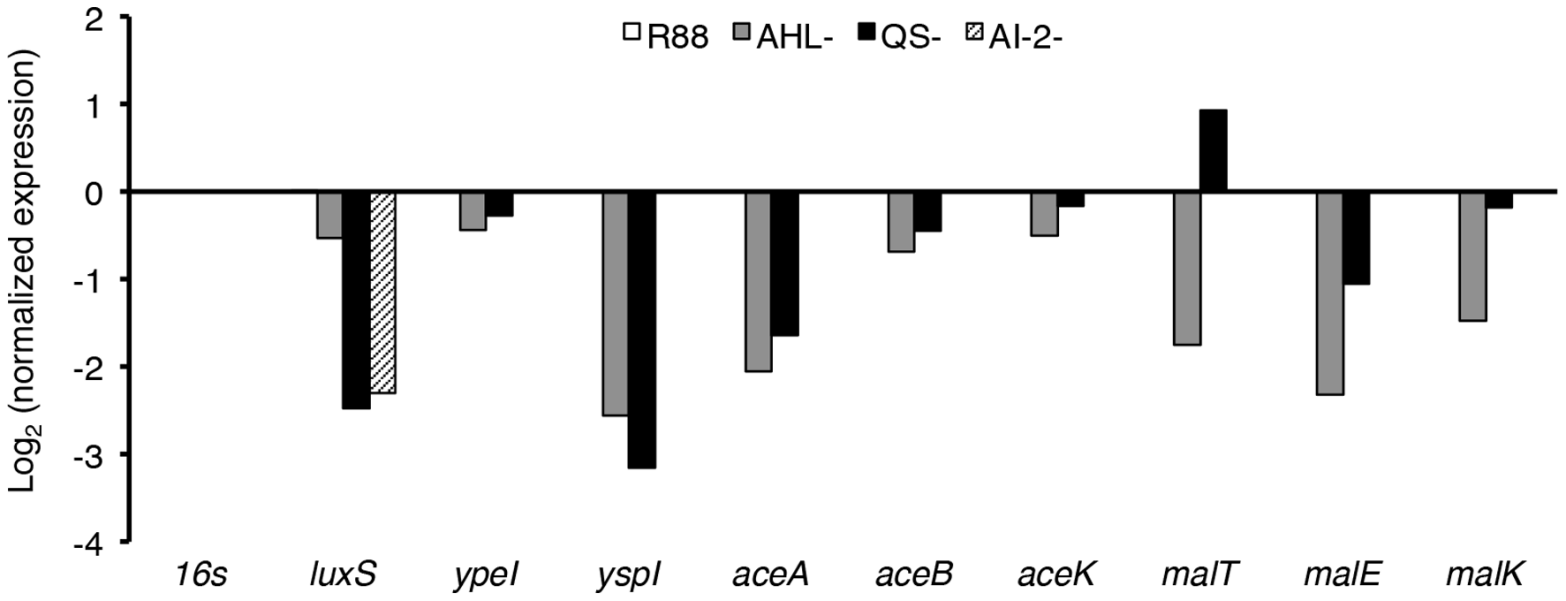
AHL-based QS regulates secondary metabolism. RNA was isolated from the indicated strains of *Y. pestis*, and levels of mRNA were measured by qRT-PCR as outlined in Material and Methods. Data represents the mean of triplicate measurements of the transcript differences between each mutant and that of strain R88 normalized to the 16 S rRNA of each sample, and are representative of at least three independent experiments. AHL^–^ = strain R114; QS^–^ = strain R115; AI-2^–^ = strain ISM1980.

The QS-mediated up-regulation of thiamine biosynthesis was dispensable *in vitro*, as the QS-null bacteria (strain R115) had no growth deficiency in thiamine-free media even after washing the cells and diluting into thiamine free media (data not shown). Likewise, there was no growth deficiency with galactose as the sole carbon and energy source (data not shown) suggesting that the *gal*-operon transcription is not completely repressed in the QS-mutant thus allowing growth with galactose. The *mal* operon, *aceA* and *aceB*, and *katY*, were confirmed by RT-PCR as being up-regulated by quorum sensing. Chen et al. showed previously by protein microarray an up-regulation in KatY by QS [31]. Further analysis revealed *mal* genes and *aceAB* are up-regulated by the Ype AHL QS system, whereas *katY* expression is responsive to AI-2 (Fig. 2).

**Figure 3:**
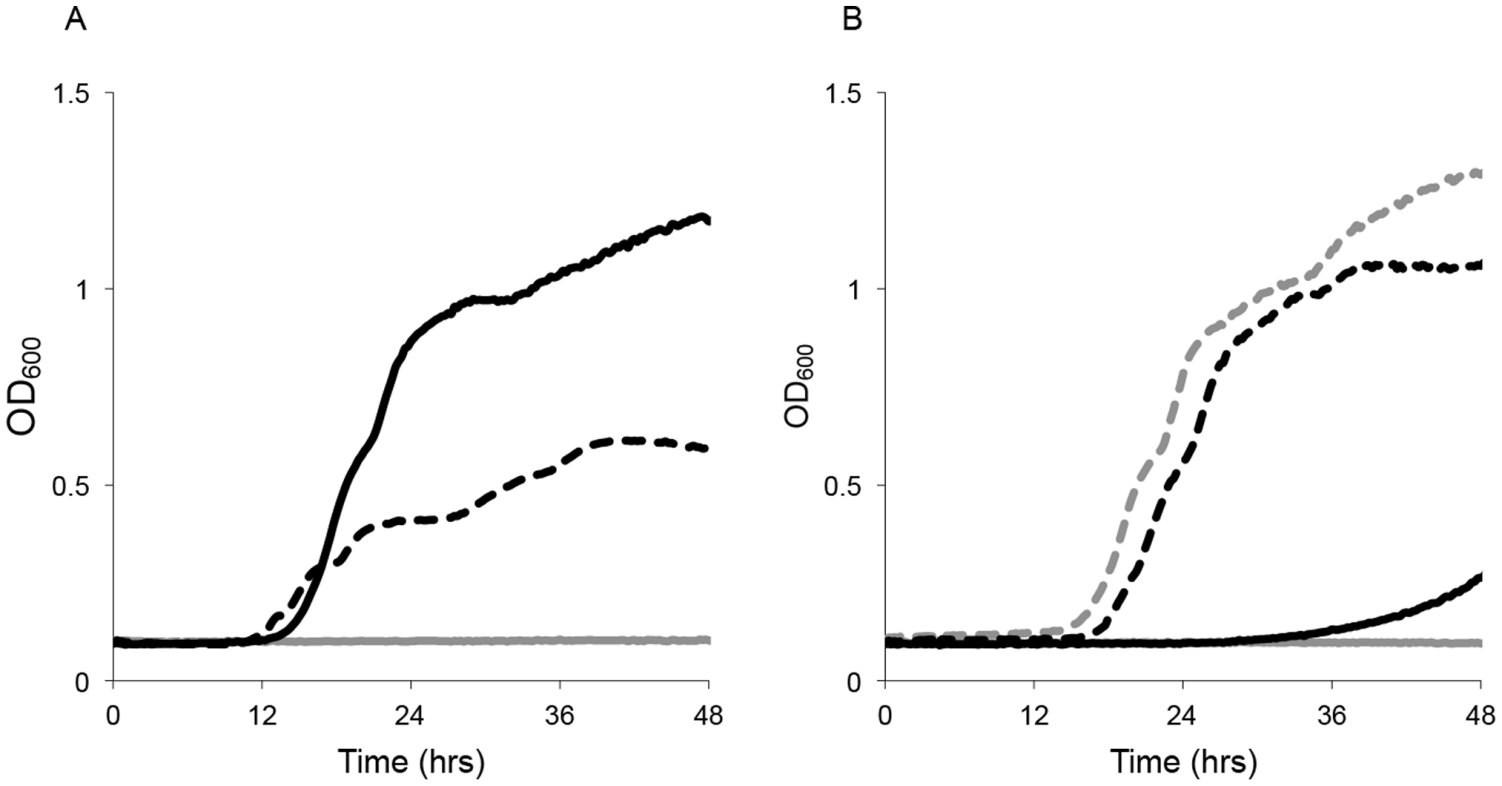
AHLs upregulate the maltose operon and enhance growth on maltose. (A) Growth of wild-type (WT) (black), and AHL mutant (dashed) *Y. pestis* in minimal maltose medium (light grey) was monitored in Bioscreen C microplate reader incubating at 28°C with agitation. (B) Complementation of AHL mutant bacteria with p*ypeIR* (black, dashed) or p*yspIR* (grey, dashed) restores growth on maltose, whereas control plasmid (black, solid) does not.

**Figure 4:**
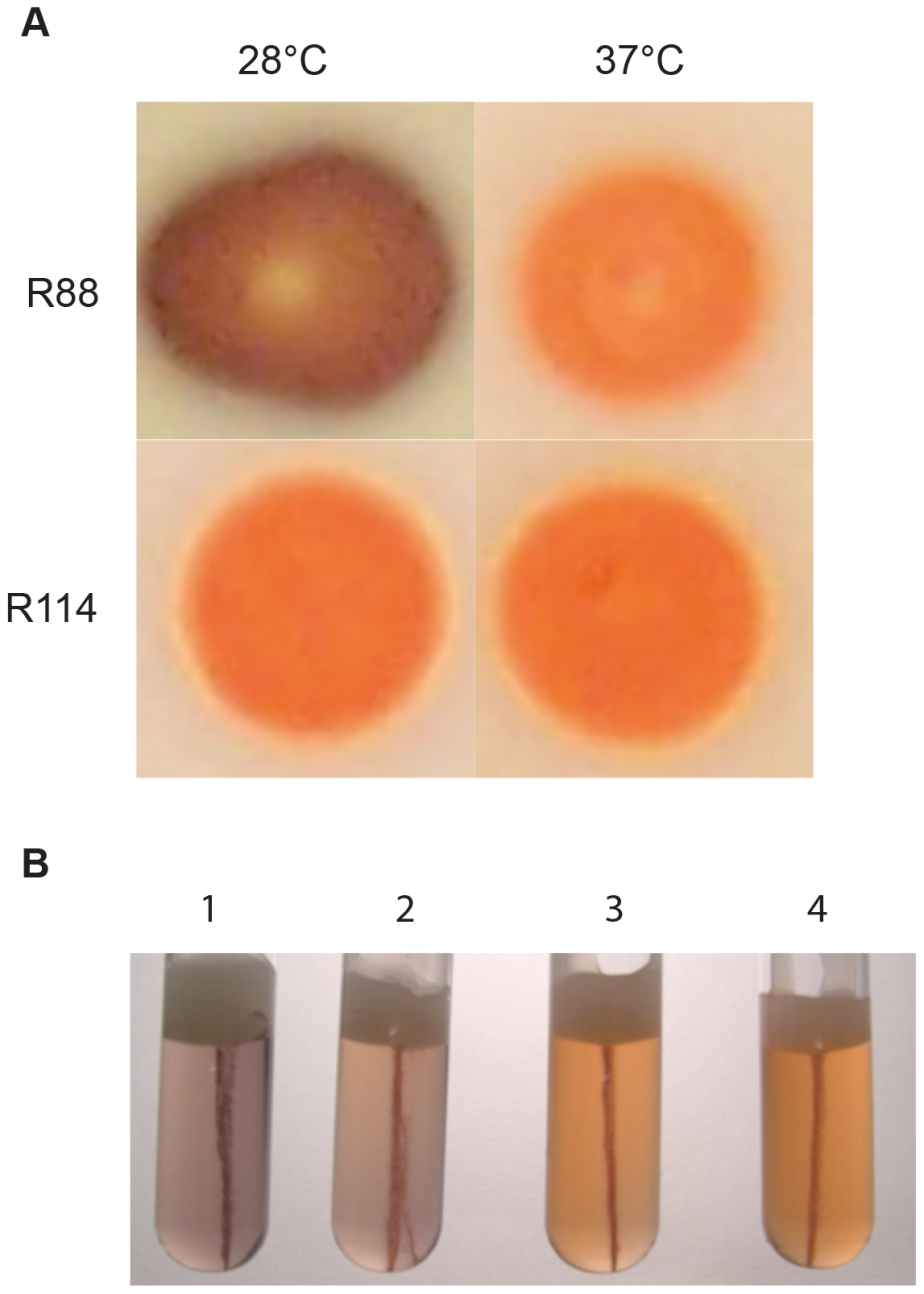
Quorum-sensing regulates fermentation of sugars. (A) Colony phenotype after 96 hrs growth on LB plates containing Congo red and 0.2% maltose and incubated at the temperature indicated. *Y. pestis* ferments maltose to acid, converting the Congo red to black, whereas a *yspIR ypeIR* mutant does not. This fermentation does not occur at temperatures above 30°C, or on other sugars tested (data not shown.) (B) Duplicate growth stabs of *Y. pestis* grown on solid medium under anaerobic conditions for 96 h at 28°C. An indicator dye, Congo Red, turns dark upon production of fermentative end products. Deletion of *yspIR* and *ypeIR* in R114 and R115 results in a lag in fermentation of maltose under anaerobic conditions. 1 = *Y. pseudotuberculosis*; 2 = R88; 3 = R114; 4 = R115.

An AHL-null *Y. pestis* (strain R114) is growth deficient with maltose as the sole carbon and energy source (Fig. 3A). Growth with other carbon sources or in rich media was not impacted by the QS mutations (data not shown). Complementation of the QS mutant strain by expressing either *ypeIR* or *yspIR* on a plasmid restored growth on maltose (Fig. 3B). During growth on LB agar plates containing maltose and Congo red, maltose growth is fermentative and results in acid production (Figure 4a). This is manifested by a dark red color change due to the Congo red in the medium. Under anaerobic conditions, the AHL-null strain still fails to ferment maltose (Figure 4b). Since previous microarray results show significant down-regulation of the *mal* operon at 37°C in LB [32], blood [33], and during rat infection [34], we decided to compare growth on maltose at each temperature. Fermentative growth was only observed in R88 grown at 28°C. At 37°C, *mal* transcription was low and *Y. pestis* failed to ferment maltose (Figure 4A, unpublished results).

### The QS-controlled Glyoxylate Bypass is QS-controlled and Required for Growth on Fatty Acid Substrates

**Figure 5:**
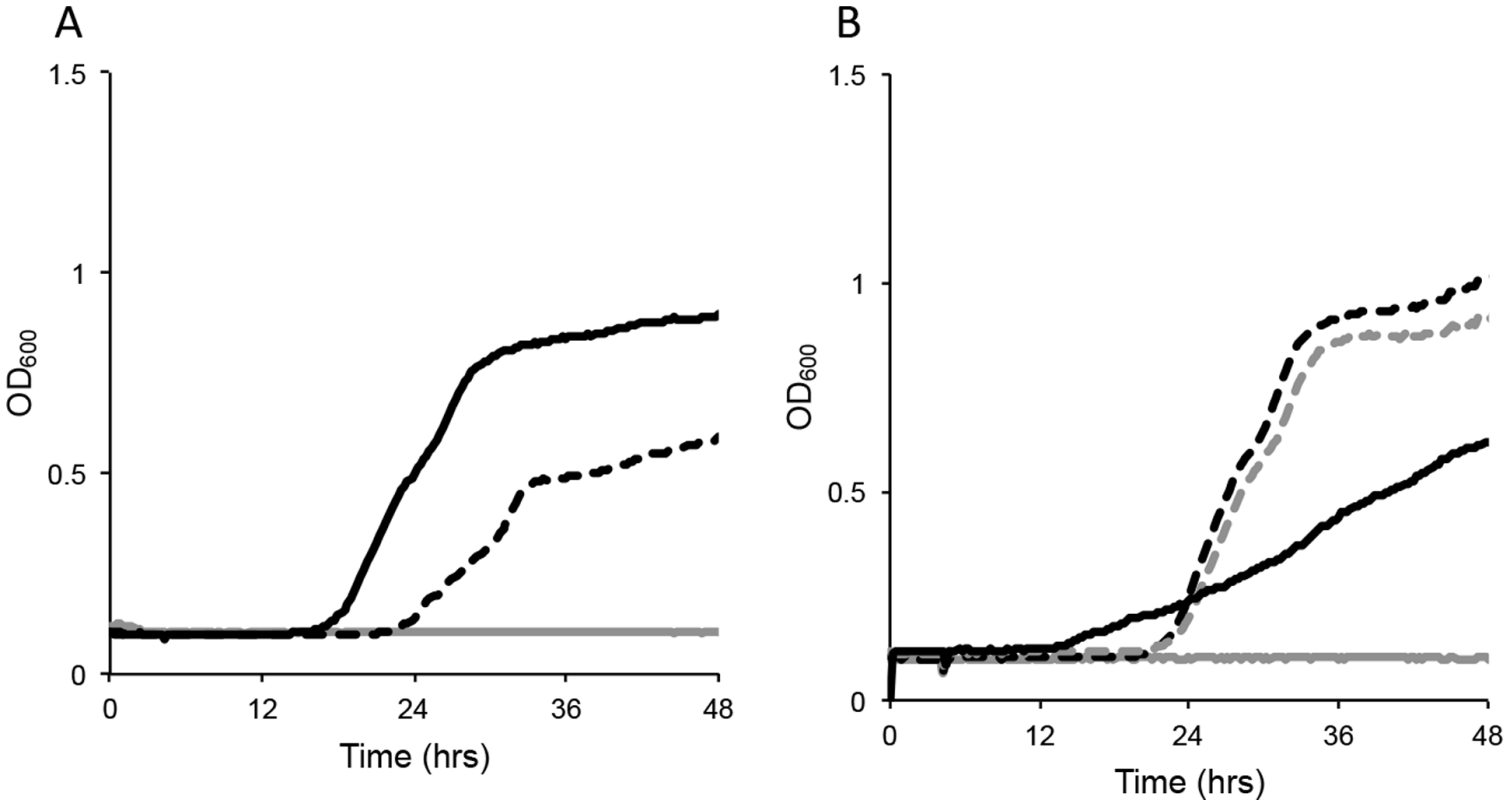
AHL quorum sensing upregulates glyoxylate bypass and enhances growth on acetate. (A) Growth of R88 (black), and an R115 AHL null mutant strain (dashed) of *Y. pestis* in minimal acetate medium (light grey is medium only control) was monitored in a Bioscreen C microplate reader incubating at 28°C with agitation. (B) Complementation of AHL mutant bacteria with p*ypeIR* (black, dashed) or p*yspIR* (grey, dashed) restores growth on acetate, whereas control plasmid (black, solid) does not.

The glyoxylate bypass of the TCA cycle is utilized during growth on fatty acids and acetate. This pathway depends on the *aceA* and *aceB* encoded enzymes, isocitrate lyase and malate synthase, to bypass the carbon dioxide evolving steps of the TCA cycle [35]. This metabolic variation on the TCA cycle is critical for pathogens growing on fatty acid substrates. Constitutive expression of *aceA* and *aceB* results in the glyoxylate bypass without induction, a trait of *Y. pestis*, that serves as a diagnostic tool [29], [36]–[38]. The *aceA* and *aceB* genes were found to be highly up-regulated by AHL signals (Table 2 and Figure 2). Diminished *aceAB* expression in the QS mutant, strain R115, at high cell densities suggested an impaired ability to grow on substrates that flow through the glyoxylate cycle, like acetate or fatty acids. Indeed, in cultures with acetate as the sole carbon and energy source, the QS null strain, R115, expresses *aceA* and *aceB* at lower levels (data not shown) and lags behind WT in growth (Fig. 5) at 30°C but not at 37°C. These results corroborate previous microarray observations that the *aceA* is relatively poorly expressed at 37°C or during exponential growth, compared to lower temperatures or stationary phase [34].

## Discussion

In this study, we demonstrate that AHL-based QS is important for the regulation of metabolism in *Y. pestis*. This represents a significant departure from the functions QS regulates in related strains within the *Yersinia* genus. This is likely a reflection of *Y. pestis*’s host-dependent lifecycle, where host-derived nutrients are the focal point of metabolism in this species. Nutritionally, *Y. pestis* can be characterized as growing primarily on blood, causing septicemia in the mammal and growing on digested blood meals within the flea. Plague can grow in the flea host for long periods, but is non-invasive being nutritionally sustained by the initial blood meal and subsequent blood meals [39]. There is a great deal of variability in susceptibility to plague for both mammalian species and the flea, but it is clear that fleas can represent a long-term reservoir for plague, some maintaining infection for over a year [3]. Likewise, the source of the blood meal influences the *Y. pestis* growth rate and infection frequency in the flea [4]. The combination of the closed environment of the flea that will rapidly become deficient in nutrients, and the necessity to survive long periods on sporadic feedings, makes the ability to fully utilize the blood meal necessary.

Previous microarray analysis has shown that *aceA* is most strongly up-regulated in the stationary phase of growth at 21°C compared to 37°C [34]. This is consistent with our own results. This regulation pattern correlated with the work in other species showing that isocitrate lyase (ICL) is most important during chronic rather than acute infection [40]. Furthermore, down-regulation at 37°C precludes infection of the mammal, further implying its importance during persistent colonization of the flea. Up-regulation of the glyoxylate bypass pathway would provide a selective advantage for growth on the fatty acids of the blood meal, and provide a way to scavenge acetate from fatty acid breakdown and earlier growth on the blood glucose. Importantly, previous assays for glyoxylate pathway activity were performed at high cell densities, when quorum-sensing regulated genes would be expected to be up-regulated. The glyoxylate bypass pathway is critical for the pathogenesis of several species. *Salmonella* ICL mutants are fully competent for growth and acute infection, but they cannot cause persistent infections [40]. Likewise, ICL is required for *Mycobacterium tuberculosis* persistence in macrophages [41], and *Pseudomonas aeruginosa* ICL mutants do not thrive in a lung model of cystic fibrosis [42]. Isocitrate lyase expression is uniquely constitutive in *Y. pestis* [36], [38], highlighting a potential adaptation of plague. While this study notes the impact of this regulation on growth *in vitro*, these are underscored by the observation that *aceA* is not required for infection following intravenous inoculation of mice, nor for infection, blockage, or growth in the flea vector [29].

As with fatty acid degradation, utilization of particular carbohydrates can be necessary for a pathogen to establish or persist during disease. Among the carbohydrates shown to be nutritionally important in streptococcal pathogenesis are mannose, maltose, galactose, and sucrose [43]. Increased maltose catabolism gives *Escherichia coli* O157:H7 a competitive advantage during colonization [44]. The maltose operon in defined media and human plasma is highly up-regulated at 26°C compared to 37°C in a growth phase-dependent manner in *Y. pestis* [32], [33]. Proteins of the maltose operon are among the most abundant proteins in stationary cells at 26°C in defined media and appear at much higher levels over cells grown at 37°C or growing logarithmically [45]. In these experiments, inducers of the maltose operon were absent, reflecting up-regulation of the maltose operon by unknown mechanisms. Significant up-regulation of the *gal* operon was observed, although QS was not required for growth on galactose. This may indicate that this regulation may be incidental or refractory to single carbon source tests and instead has a different role in bacterial physiology. An explanation for the potential benefit of *gal* induction may lay in the observation that the *gal* and *mal* operons, and *aceAB* of *E. coli* are induced by glucose limitation during continuous culture [46], suggestive of additional roles in cell metabolism. Induction of these pathways increases glucose affinity [46], and mutations resulting in *gal*, *mal*, and *aceAB* up-regulation are commonly found during long-term culture experiments and during infections [46].

Why regulation of secondary carbon and energy source pathways is subject to quorum sensing control in plague is unclear. We speculate that QS may coordinate the use of alternative growth substrates in the flea. After a blood meal, a period of rapid growth on a primary carbohydrate of blood, glucose, may produce a “quorum” of *Yersinia*. Following depletion of glucose, alternative growth substrates present in blood would then be utilized. This strategy might optimize the growth yield of *Y. pestis* in the flea environment or help to sustain it under limiting growth conditions.

## Supporting Information

Table S1 **Primers used in RT-PCR experiments.**
(XLSX)

Table S2 Genes significantly expressed by quorum sensing (*p*<0.05, *q* <0.08, FC >1.5). Strains R88 CO92 Δ*pgm* pCD^+^ pMT1^+^ pPCP+ and R114 (CO92 Δ*pgm* pCD^+^ pMT1^+^ pPCP^+^ Δ*ypeIR* Δ*yspIR*) were compared.
(XLSX)
