# Correction: Transcriptome Analysis of Acyl-Homoserine Lactone-Based Quorum Sensing Regulation in *Yersinia pestis*

**DOI:** 10.1371/annotation/3f0e8e82-1db8-45fe-a700-c64ede3e3c61

**Published:** 2013-09-10

**Authors:** Christopher N. LaRock, Jing Yu, Alexander R. Horswill, Matthew R. Parsek, F. Chris Minion

The word "Acyl" was spelled incorrectly in the title. The correct title is: Transcriptome Analysis of Acyl-Homoserine Lactone-Based Quorum Sensing Regulation in *Yersinia pestis*.

The correct citation is: LaRock CN, Yu J, Horswill AR, Parsek MR, Minion FC (2013) Transcriptome Analysis of Acyl-Homoserine Lactone-Based Quorum Sensing Regulation in *Yersinia pestis*. PLoS ONE 8(4): e62337. doi:10.1371/journal.pone.0062337 

